# Pregnancy, Labor, and the Puerperal State

**Published:** 1896-02

**Authors:** 


PREGNANCY, LABOR, AND THE PUERPERAL STATE. By Egbert H. Grandin, M. D., Consulting Surgeon to the New York Maternity Hospital; Consulting Gynæcologist to the French Hospital, N. Y., etc.; and George W. Jarman, M. D., Obstetric Surgeon to the New York Maternity Hospital, Gynæcologist to the Cancer Hospital, N. Y., etc. Illustrated with forty-one original full-page photographic plates from nature. Royal octavo, pages viii, 261. Cloth, $2.50 net. Philadelphia: The F. A. Davis Co., Publishers, 1914 and 1916 Cherry Street.

In THE HOMŒOPATHIC PHYSICIAN for March, 1895, at page 146, was given a review of a book on Obstetric Surgery, by Egbert H. Grandin, M. D., and George W. Jarman, M. D. These authors have now supplemented that work with another on the whole subject of obstetrics from the physiological side, and that is the book now under notice. Such a book is needed. There are so many improvements and so much generally that is new that a book which has been brought up to date is very acceptable. The present work is illustrated by photographs. The whole mechanism of labor is graphically explained and illustated with these photographs.

It is divided into three parts—Pregnancy, Labor, and the Puerperal State. In the first part, Pregnancy, are treated the diagnosis of pregnancy, the pathology of pregnancy, and the diagnosis of the presentation of the fœtus. In the second part, Labor, the mechanism, clinical course of labor, management of labor, normal and abnormal, and care of the new-born infant are treated, while in the third part, the Puerperal State, the normal and the pathological conditions are treated. The book finishes with an index. It is uniform in size with the *Surgery* before mentioned, and is intended as an accompaniment to it, making thus a complete survey of the whole subject.

The unusually cordial reception extended to the *Obstetric Surgery* has greatly stimulated both authors and publishers to bring out the companion volume on *Pregnancy, Labor, and the Puerperal State*, and thus place in the hands of medical students and practitioners a thoroughly modern text-book on this very important branch of medical and surgical science. The two volumes are now bound together in one, thus forming a compact and handsome volume, covering the entire field of obstetric science.

More than fifty medical colleges have within a few months adopted it as their principal text-book on this subject; thus the work has met the needs of the majority of the ablest clinical teachers as *the* book of real service and value from which to teach—and learn—the science and art of midwifery.

